# Association Between Chronic Pain and Fatigue Severity with Weather and Air Pollution Among Females with Myalgic Encephalomyelitis/Chronic Fatigue Syndrome (ME/CFS)

**DOI:** 10.3390/ijerph21121560

**Published:** 2024-11-26

**Authors:** Chloe Lisette Jones, Olivia Haskin, Jarred Wayne Younger

**Affiliations:** Department of Psychology, The University of Alabama at Birmingham, Birmingham, AL 35233, USA; och@uab.edu (O.H.); youngerlab@uab.edu (J.W.Y.)

**Keywords:** chronic pain, fatigue, myalgic encephalomyelitis, air quality, weather

## Abstract

Weather and air quality conditions have been anecdotally reported to be related to symptom fluctuations in Myalgic Encephalomyelitis/Chronic Fatigue Syndrome (ME/CFS), but this has never been empirically investigated. This exploratory study aims to examine the effects of weather and air quality on daily fluctuations of chronic pain and fatigue in women with ME/CFS. In an intensive longitudinal design, 58 participants with ME/CFS provided daily pain and fatigue ratings for an average of 61 days. Daily weather and air quality data were obtained from the National Oceanic and Atmospheric Administration and the US Environmental Protection Agency for the Birmingham, AL area. Linear mixed models revealed a significant relationship between days with more severe pain and worse Air Quality Indices (AQI, *p* < 0.001), lower wind speeds (*p* = 0.009), greater particulate matter (*p* = 0.037), and lower carbon monoxide (*p* = 0.004), sulfur dioxide (*p* = 0.003), and ozone levels (*p* = 0.015). Greater fatigue was associated with more particulates (*p* = 0.023) and lower barometric pressure (*p* = 0.048). These results suggest that air quality and weather can have small effects on ME/CFS symptom severity.

## 1. Introduction

Myalgic Encephalomyelitis/Chronic Fatigue Syndrome (ME/CFS) is a disabling condition characterized by unexplained, long-term fatigue that is not alleviated by rest, and often accompanied by impaired memory or concentration, sore throat, tender lymph nodes, muscle pain, multi-joint pain, headaches, unrefreshing sleep, and the worsening of symptoms following exertion (i.e., Post-Exertional Malaise, PEM) [1]. ME/CFS was estimated in 2015 by the Centers for Disease Control (CDC) to affect as many as 836,000 to 2.5 million people in the United States, with rates disproportionately higher in women [2], though this number is thought to have increased since the onset of the COVID-19 pandemic.

As no FDA-approved treatments currently exist for ME/CFS, patients may rely on eclectic approaches to managing their symptoms, including behavioral and environmental strategies such as pacing of activities [3] or avoiding environmental triggers [4,5]. One set of possible triggers reported anecdotally by patients is weather and air quality. Although not studied in ME/CFS, symptom exacerbations with weather patterns have been documented in other pain and fatigue conditions. Fibromyalgia is a chronic pain condition which affects approximately 1.75% of the US population [6] and is frequently comorbid with ME/CFS [7]. Some research has suggested that fibromyalgia symptomatology fluctuates with weather changes. In longitudinal studies that tracked daily symptoms and weather parameters, positive associations between pain and relative humidity were identified in fibromyalgia patients [8], while associations have been found between greater fibromyalgia pain, lower barometric pressure [9,10], and lower sunshine duration [8]. In a heterogenous patient sample of various rheumatological conditions, including fibromyalgia, barometric pressure was also found to be negatively associated with self-reported joint pain over the course of four weeks [11]. Changes in temperature and precipitation have also been associated with worsening arthritic pain [11].

There are fewer studies examining the relationship between weather and fatigue. Higher temperatures have been associated with greater reported fatigue in healthy subjects, but only in those with poor sleep [12]. Fatigue in fibromyalgia was greater on days with relative higher humidity and higher temperature on the previous day [8]. The small body of research examining pain, fatigue, and weather has generally shown mixed and marginally significant effects. Another trend amongst the limited studies, however, is a relatively short study duration, small sample size, and heterogenous evaluation of weather parameters and symptoms [13].

Both acute and chronic exposure to air pollutants has been linked to deleterious health effects such as cardiovascular disease, respiratory disease, and immune system disruption [14]. The primary mechanisms of these health effects are oxidative stress and proinflammatory responses induced by particulate matter and gaseous pollutants [15,16]. Although no specific etiopathologies have been established in ME/CFS, inflammation is thought to play a critical role in the illness and account for some of the significant fluctuations in symptoms [17,18,19]. Additionally, worsening of oxidative stress may aggravate symptoms due to the hypothesized role of altered redox pathways in ME/CFS [20,21], and the relationship between oxidative stress and fatigue [22]. Therefore, minor environmental insults, such as air pollutants, may be related to daily changes in symptom severity in ME/CFS, but this has yet to be investigated.

This exploratory study aims to perform the first study of the effects of weather on chronic pain and fatigue in women with ME/CFS. To determine the association between symptoms, weather, and air quality parameters, this study employs an intensive longitudinal design with daily symptom reports. Although analyses are exploratory in nature, it is hypothesized that days with worse air quality will be associated with worse symptom severity. It is also hypothesized, as suggested by prior research in rheumatological conditions, that lower barometric pressure and higher precipitation will be associated with worse symptom severity.

## 2. Materials and Methods

### 2.1. Study Procedures

The present study includes data from a parent study evaluating fluctuations in ME/CFS symptoms and peripheral biomarkers in the blood using the same design as described in Stringer et al. [23]. Weather and air quality data were collected post-hoc after study completion and participants were therefore blind to the study aims.

Participants were recruited from the Birmingham, Alabama area. To be included, participants were required to be female, between the ages of 18 and 65, and meet the CDC criteria for ME/CFS. Because ME/CFS disproportionately affects women, only female participants were enrolled due to the difficulty of recruiting males with ME/CFS and obtaining balanced groups. The CDC diagnostic criteria for ME/CFS includes fatigue lasting more than 6 months and at least four of the following symptoms: impaired memory or concentration, sore throat, tender cervical or axial lymph nodes, muscle pain, multi-joint pain, headaches of a new type or pattern, unrefreshing sleep, and post-exertional malaise. These symptoms also cannot be explained by any other cause based on the patient’s history, physical examinations, and other diagnostic tests [1]. Exclusionary criteria included any rheumatological or autoimmune disorders, opioid use, major depressive disorder with melancholic features, acute infections, use of anti-inflammatory medications, pregnancy, positive anti-nuclear antibody test, positive rheumatoid factor test, or baseline erythrocyte sedimentation rate of >60 mm/hr. After meeting initial eligibility via phone-screening, participants attended an in-person screening session to confirm eligibility at the Clinical Research Unit at the University of Alabama at Birmingham (UAB). To confirm eligibility, participants were administered the Mini International Neuropsychiatric Interview psychological screener. Participants were also asked to provide their average pain and fatigue rating from 0 to 10 with higher numbers indicating greater severity.

All individuals consented prior to participation and study procedures were approved by the UAB Institutional Review Board. Eligible participants were provided with a touch-screen tablet to complete daily symptom reports. The first participants enrolled (*n* = 28) completed a one-week baseline period of symptom reports and then returned for daily blood draws over 25 consecutive days while providing daily symptom reports. To decrease participant burden, the study design was altered to allow participants to provide two blood samples per week for 12.5 weeks to achieve the total 25 blood draws. Therefore, the remaining participants enrolled (*n* = 30) provided daily symptom reports for 12.5 weeks. The baseline period of symptom reports was collected to ensure participants reported an average daily fatigue of at least 40 out of 100 and to ensure adherence to daily symptom reports prior to collecting blood samples. Participants were enrolled on a rolling basis and therefore data collection periods were not restricted to any specific season.

### 2.2. Measures

Participants were asked to rate several symptoms daily, including pain, fatigue, mood, stress, gastrointestinal problems, mental clarity, headaches, as well as their sleep satisfaction, satisfaction with life, functional limitations, and changes in medication. Self-reported pain and fatigue were selected as the primary outcomes for the present analyses as they represent common ME/CFS symptoms and were highly endorsed in the sample. Participants reported daily pain and fatigue on a 0–100 visual analog scale with higher numbers indicating more severe symptoms with the following questions: “How fatigued have you been over the day?” and “How would you rate your general level of pain today?”

Readings of maximum temperature (°F), minimum temperature (°F), average temperature (°F), relative humidity (%), precipitation (in), atmospheric pressure (inHg), and average wind speed (mph) were taken from the Birmingham Airport, Alabama, US station from the Local Climatological Data from the US Department of Commerce’s National Oceanic and Atmospheric Administration. Secondly, Air Quality Index Daily Value Reports from the US Environmental Protection Agency were collected for the Birmingham-Hoover, AL core-based statistical area for quantifications of major air pollutants: carbon monoxide (ppm), sulfur dioxide (ppm), nitrogen dioxide (ppm), ground-level ozone (ppm), and particulate matter 10 (PM_10_) and 2.5 (PM_2.5_) micrometers or less in diameter (micrograms/cubic meter).

### 2.3. Statistical Procedures

All statistical procedures were conducted with IBM SPSS (Version 28). A linear mixed model was created for each of the two outcomes, fatigue and pain. In each model, the day was selected as the repeated measures index and the subject identification number was entered as the repeated measures nesting variable. All weather variables were entered as continuous predictors. An autoregressive covariance type was used. Statistical significance was set at *p* < 0.05. Due to the assumed relationship between many of the weather and air quality parameters, variance inflation factors (VIF) were computed for each predictor to screen for multicollinearity in the model with a VIF threshold of 5.0. Additionally, a bivariate correlation between the dependent variables, pain and fatigue ratings, was performed.

Descriptive statistics were conducted to describe the sample characteristics. Mean and standard deviations were computed for participant age and daily average pain and fatigue ratings. Frequencies of participants’ gender, race, ethnicity, education, marital status, and work status were tabulated.

## 3. Results

### 3.1. Demographics

Baseline participant characteristics are listed in Table 1. The sample was white (*n* = 45, 77.60%), non-Hispanic/Latino (*n* = 53, 91.40%), married (*n* = 30, 51.70%), working (*n* = 29, 50.00%), with partial college education (*n* = 20, 34.50%), and was 43.14 years old on average (SD = 10.22). During initial screening procedures, participants reported an average fatigue level of 6.90 (SD = 1.62) and an average pain level of 5.00 (SD = 2.38) on a 10-point scale, with higher scores indicating higher severity. The participants who provided daily symptom reports for 25 consecutive days and a baseline period reported their symptoms for a total of 37.82 days on average (SD = 10.22). The participants who provided daily symptom reports for 12.5 weeks and a baseline period reported their symptoms for a total of 84.33 days on average (SD = 16.44). The combined sample (*n* = 58) provided daily symptom reports for an average of 61.88 days (SD = 27.14).

### 3.2. Weather and Reported Symptoms

#### 3.2.1. Variance Inflation Factors

There were three predictors with a variance inflation factor greater than 5.0. As expected, average temperature, maximum temperature, and minimum temperature were strongly related with variance inflation factors of 101.37, 45.17, and 27.15, respectively. The remaining predictors all had variance inflation factors of <5.0 and therefore did not suggest multicollinearity. When maximum and minimum temperatures were removed from the model, average temperature was no longer multicollinear with any other predictor. Therefore, maximum and minimum temperature were removed from all further analyses, while average temperature was retained. Bivariate correlations revealed that daily pain and fatigue ratings were significantly positively correlated as expected, with a coefficient of 0.43 (*p* < 0.001).

#### 3.2.2. Fatigue

To determine if there was a relationship between weather and fatigue in women with ME/CFS, the weather data and the participants’ self-reports of fatigue were analyzed via a linear mixed model. This analysis revealed a marginally significant inverse relationship between reported daily fatigue and atmospheric pressure F(1, 2407.06) = 3.92, *p* = 0.048), while levels of PM_10_ were significantly positively associated with daily fatigue levels (F(1, 2670.85) = 5.18, *p* = 0.023). The other weather and air quality predictors did not show any significant relationship with fatigue in women with ME/CFS.

#### 3.2.3. Pain

**Figure 1:**
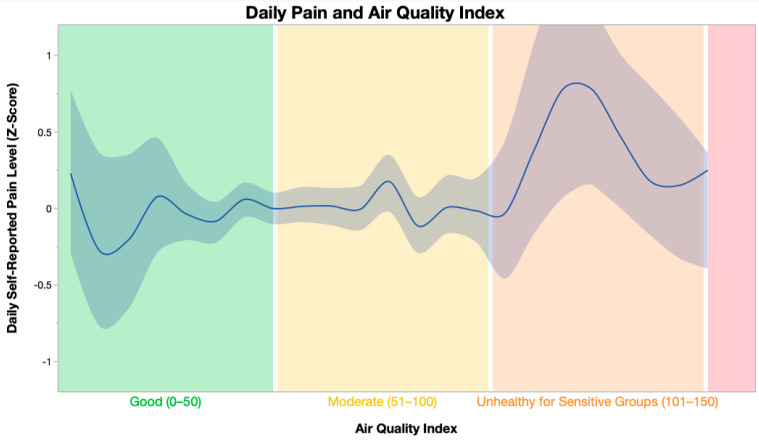
Daily self-reported pain and the Air Quality Index. The Air Quality Index ranges are categorized by level of concern (Good, Moderate, and Unhealthy for Sensitive Groups) and associated with a specific color (Green, Yellow, Orange) in accordance with the United States Environmental Protection Agency Air Quality Index reporting.

Self-reported average daily pain was significantly positively related to the Air Quality Index (F(1, 2636.61) = 22.75, *p* < 0.001) (Figure 1), PM_10_ (F(1, 2669.50) = 4.374, *p* = 0.037), and inversely related to wind speed (F(1, 2668.15) = 6.76, *p* = 0.009), carbon monoxide (F(1, 2673.91) = 8.38, *p* = 0.004), sulfur dioxide (F(1, 2644.41) = 8.71, *p* = 0.003), and ozone (F(1, 2673.58) = 5.98, *p* = 0.015). Average temperature, precipitation, pressure, humidity, nitrogen dioxide, and PM_2.5_ were not significantly related to pain. The results of the linear mixed models can be seen in Table 2 and Table 3.

Description of Air Quality Index ranges on the *x*-axis were obtained from the United States Environmental Protection Agency. Daily self-reported pain levels on the *y*-axis were person-centered to minimize the effect of interindividual variability in pain ratings. The band represents the 95% confidence interval.

## 4. Discussion

Pain exacerbations due to weather have long been reported in arthritic conditions, usually implicating changes in atmospheric pressure, humidity, and temperature. The literature, however, has only identified small to moderate and mixed effects of weather in this population [15,16]. Further analyses have suggested that certain subgroups of patients may be differentially sensitive to changes in weather patterns [8,11,24], and that those with greater sensitivity to weather changes may show lower quality of life [25].

Unlike prior studies involving chronic pain conditions, pain was not significantly associated with barometric pressure or precipitation. In the present study, worse pain was associated with overall worse air quality (AQI), lower wind speeds, and more particulate matter (PM_10_). Unexpectedly, greater carbon monoxide (CO) levels were associated with lower levels of pain. Interestingly, this inverse relationship has also been observed in sickle cell disease patients, with lower levels of atmospheric CO being associated with increased hospital admissions for acute pain crises [26,27,28]. The mechanism underlying the relationship between CO and pain in sickle cell disease, however, likely diverges from that which may underlie CO and pain in ME/CFS. Similarly, sulfur dioxide (SO_2_) and ozone (O_3_) were also inversely related to pain in the present study. Nitrogen dioxide (NO_2_) and fine particles (PM_2.5_) were not related to pain. It is notable that during this study period, the pollutant levels were generally considered acceptable. Studies including higher pollutant levels might find different or additional relationships.

In this study period, fatigue ratings were worse on days with higher levels of particulate matter (PM_10_) and lower barometric pressure. This is the first observation, to our knowledge, of an association between barometric pressure and fatigue. A study of healthy traffic officers in 2015 also found higher levels of PM_10_ to be associated with greater fatigue [29], suggesting that this relationship may not be illness-specific; however, it is possible that certain populations may be more sensitive to the effects of pollution.

Although the illness has no widely accepted etiology, it has been theorized that ME/CFS is a syndrome representing low-level, chronic, neuroinflammatory processes [30,31]. Long-term exposure to heavy air pollution has been associated with neuroinflammatory processes, including disruption of the blood brain barrier, oxidative stress, epithelial and endothelial barrier damage, and altered innate immune responses [32,33]. Air pollutants have also been identified as disrupting neuroendocrine stress responses [34], impacting central nervous system function [35]. Therefore, the finding in the present study may be partly explained by air pollutants exacerbating pathological neuroinflammatory processes occurring in ME/CFS. Although the present results should be interpreted with caution, if poor air quality worsens ME/CFS symptom severity, several mitigating strategies could be identified to offset the negative multisystem effects. For example, since air pollutants lower vitamin D synthesis [36], vitamin D supplementation may be considered a potential mitigating effort, or microbiome interventions to offset negative impacts on gut health [37], along with efforts to improve surrounding air quality. To improve ambient air quality, the US EPA recommends identifying and eliminating indoor sources of pollution and improving ventilation as primary measures. To reduce the harmful health effects of pollution, the CDC’s National Center for Environmental Health currently recommends monitoring the Air Quality Index and avoiding outdoor activity, and particularly outdoor exercise, on days with poor air quality.

In this study, several significant relationships were identified between symptoms in ME/CFS and weather and air quality factors. With the exception of the relationship between the AQI and pain, the majority of these effects were small, as observed in prior literature on other chronic conditions. Other factors, including physical exertion, are presumed to account for a greater amount of the variance in ME/CFS symptoms [38,39]. It is possible, however, that for ME/CFS patients, adopting a combination of strategies that may have individually small-to-moderate effects could still cumulatively contribute to an impactful decrease in symptoms and an increase in functioning, though this cannot be ascertained from the current study.

### Limitations

The present study has several limitations. All participants were recruited from the Birmingham, AL area, and all meteorological data were also restricted to this geographic area. This limits the generalizability of these findings to other areas with vastly different climate conditions. A benefit of this restricted catchment area, however, is that other meteorological or climate influences were also kept constant for all participants in the study. Additionally, this study only included female participants. Although the majority of individuals with ME/CFS are women, the illness afflicts both men and women. Therefore, the findings of this study may not be generalizable to men with ME/CFS. Decentralized designs that allow for larger and more diverse patient samples are recommended for future investigations.

This study is also limited in that the amount of time participants spent per day outside was not collected or estimated. Based on the overall study design, however, participants were required to visit the study site on many days throughout their participation. Therefore, despite individual variability in outdoor exposure, at a minimum, all participants were exposed to the study site surrounding air quality and weather conditions on a regular basis throughout the collection period.

The size of the sample precluded any subgroup analysis; some research has suggested that weather sensitivity may differ among individuals [8,11,24,25,40,41]. Future research may examine potential subgroups and interindividual differences in weather and pollution sensitivities. Additionally, without a control group, we cannot contrast or compare the effects of pollution and weather parameters on healthy individuals to individuals with ME/CFS.

Lastly, it is difficult to disentangle the multidirectional relationships between weather, pollution, fatigue, pain, and other psychological factors. Some investigators have suggested that the relationship between weather and symptoms is better explained by a patient’s belief in weather sensitivity, rather than by weather itself [42]. In the present study, participants were blinded to the study objectives, and this is therefore considered unlikely. Nevertheless, it is recommended that psychological factors, such as belief in weather sensitivity and changes in mood, be considered in future research.

## 5. Conclusions

This study was the first to assess the relationship between weather, air quality, and ME/CFS symptoms. Of the 14 different variables analyzed in this study, seven were significantly related to pain or fatigue in women with ME/CFS. Overall, self-reported pain showed stronger associations with weather and pollution compared to fatigue. The strongest association observed in this sample was that of worse air quality and higher self-reported pain as measured by the global Air Quality Index (AQI), though overall the observed effects were small. These data can contribute further insight into potential mechanisms of exacerbating and mitigating factors in ME/CFS.

## Figures and Tables

**Table 1:** Sample characteristics.

Characteristic		*n*	%	M	SD
Average Daily Fatigue				6.90	1.62
Average Daily Pain				5.00	2.38
Age				43.14	10.22
Gender	Women	58	100.00		
	Men	0	0.00		
Race	Asian/Pacific Islander	3	5.20		
	Black, African American	8	13.80		
	White	45	77.60		
	Other	2	3.40		
Ethnicity	Hispanic or Latino	4	6.90		
	Non-Hispanic or Latino	53	91.40		
Education	Graduate/Professional Degree	16	27.60		
	Standard College Degree	15	25.90		
	Partial College	20	34.50		
	High School Diploma/GED	6	10.30		
	Less than High School	1	1.70		
Marital Status	Single	16	27.60		
	Married	30	51.70		
	Divorced	7	12.10		
	Widowed	4	6.90		
Work Status	Working	29	50.00		
	Unemployed	8	13.80		
	Student	2	3.40		
	On Disability	7	12.10		
	Homemaker	7	12.10		
	Other	2	3.40		

**Table 2:** Estimates of effects of weather parameters on fatigue.

Parameter	Estimate	Std. Error	df	t	Sig.
Average Temperature	0.031	0.023	2181.422	1.341	0.18
Precipitation	−0.008	0.023	2608.283	−0.361	0.718
Pressure	−0.043	0.022	2407.062	−1.98	0.048
Wind Speed	−0.014	0.026	2672.477	−0.544	0.587
Humidity	0.045	0.026	2657.742	1.72	0.086
Air Quality Index (AQI)	0.041	0.033	2661.36	1.241	0.215
Carbon Monoxide (CO)	−0.031	0.024	2661.945	−1.303	0.193
Sulfur Dioxide (SO_2_)	−0.003	0.022	2666.22	−0.137	0.891
Nitrogen Dioxide (NO_2_)	−0.014	0.027	2629.763	−0.509	0.611
Ozone (O_3_)	0.00	0.028	2657.885	−0.006	0.995
PM_10_	0.055	0.024	2670.849	2.276	0.023
PM_2.5_	−0.035	0.033	2565.638	−1.081	0.28

**Table 3:** Estimates of effects of weather parameters on pain.

Parameter	Estimate	Std. Error	df	t	Sig.
Average Temperature	0.000	0.024	2379.886	0.00	1.00
Precipitation	0.042	0.023	2567.985	1.85	0.064
Barometric Pressure	−0.015	0.022	2555.828	−0.68	0.497
Wind Speed	−0.068	0.026	2668.15	−2.601	0.009
Humidity	0.039	0.026	2629.705	1.506	0.132
Air Quality Index (AQI)	0.158	0.033	2636.605	4.769	<0.001
Carbon Monoxide (CO)	−0.069	0.024	2673.91	−2.895	0.004
Sulfur Dioxide (SO_2_)	−0.066	0.022	2644.413	−2.952	0.003
Nitrogen Dioxide (NO_2_)	−0.042	0.027	2589.815	−1.553	0.121
Ozone (O_3_)	−0.069	0.028	2673.584	−2.444	0.015
PM_10_	0.051	0.024	2669.504	2.091	0.037
PM_2.5_	−0.048	0.033	2634.882	−1.459	0.145

## Data Availability

The data presented in this study will be made available by the authors on request.
